# Phytotoxic Mechanism of Nanoparticles: Destruction of Chloroplasts and Vascular Bundles and Alteration of Nutrient Absorption

**DOI:** 10.1038/srep11618

**Published:** 2015-06-25

**Authors:** Le Van Nhan, Chuanxin Ma, Yukui Rui, Shutong Liu, Xuguang Li, Baoshan Xing, Liming Liu

**Affiliations:** 1 College of Resources and Environmental Sciences, China Agricultural University, Beijing 100193, China; 2 Research Institute for Aquaculture No.1, Tu Son, Bac Ninh 222260, Vietnam; 3 Stockbridge School of Agriculture, University of Massachusetts, Amherst, MA 01003, USA

## Abstract

This study focused on determining the phytotoxic mechanism of CeO_2_ nanoparticles (NPs): destroying chloroplasts and vascular bundles and altering absorption of nutrients on conventional and Bt-transgenic cottons. Experiments were designed with three concentrations of CeO_2_ NPs including: 0, 100 and 500 mg·L^−1^, and each treatment was three replications. Results indicate that absorbed CeO_2_ nanoparticles significantly reduced the Zn, Mg, Fe, and P levels in xylem sap compared with the control group and decreased indole-3-acetic acid (IAA) and abscisic acid (ABA) concentrations in the roots of conventional cotton. Transmission electron microscopy (TEM) images revealed that CeO_2_ NPs were absorbed into the roots and subsequently transported to the stems and leaves of both conventional and Bt-transgenic cotton plants via xylem sap. In addition, the majority of aggregated CeO_2_ NPs were attached to the external surface of chloroplasts, which were swollen and ruptured, especially in Bt-transgenic cotton. The vascular bundles were destroyed by CeO_2_ nanoparticles, and more damage was observed in transgenic cotton than conventional cotton.

Nanomaterials have been applied in many aspects of life processes and products, including wastewater treatment, food processing, catalytic materials, and biomedical products^1^,^2^,^3^,^4^. They are used in agriculture to enhance seed germination and plant growth and to protect crops from biotic stresses, such as insects, fungi, and bacteria^5^,^6^,^7^,^8^. However, given the unique properties of nanoparticles (NPs), living organisms in the ecosystem could suffer from oxidative stress induced by NPs^9^,^10^,^11^. A common conclusion from recent studies was that the nanotoxicity might depend on either metal speciation or the plant species. Ma *et al.*^12^ reported that exposure to 2000 mg·L^−1^ NPs from the lanthanide series, such as lanthanum oxide (La_2_O_3_), gadolinium oxide (Gd_2_O_3_), and ytterbium oxide (Yb_2_O_3_), greatly inhibits root growth in radish, tomato, rape, lettuce, wheat, cabbage, and cucumber plants. According to Lin and Xing^13^, a concentration of 200 mg·L^−1^ of nano-sized Zn (zinc, 35 nm) and ZnO (zinc oxide, 20 nm) inhibited germination in ryegrass and corn, respectively. The root growth of radish, rape canola, ryegrass, lettuce, corn, and cucumber species was also inhibited upon exposure to 2000 mg·L^−1^ nano-sized Zn and ZnO^13^. Silicon oxide NPs (SiO_2_) notably inhibit plant growth and the shoot and root biomasses of *Bacillus thuringiensis* (Bt)-transgenic cotton^14^. Le *et al.*^14^ reported that the height, shoot biomass, and root biomass of Bt-transgenic cotton were 65.39%, 68.55%, and 57.69%, respectively, of those of the control group when exposed to 2000 mg·L^−1^ of SiO_2_ NPs. Furthermore, Li *et al.* found that cerium oxide (CeO_2_) NPs disrupt the uptake of nutrient elements in Bt-transgenic cotton compared with its parental conventional cotton plant^15^. Thus, the phytotoxic mechanism of nanomaterials must be thoroughly understood before application in fields.

Transgenic plants offer numerous advantages to producers, consumers, and the environment. Indeed, engineered plants are highly tolerant to pesticides and herbicides, demonstrate resistance towards pathogens caused by microorganisms, and exhibit increased production yields and enhanced biofuel content, which are considered novel and sustainable energy sources^16^. Bt-transgenic cotton contains one foreign gene derived from the bacterium *Bacillus thuringiensis* that encodes the Cry toxin, which effectively kills bollworms and therefore controls yield losses^17^,^18^,^19^. In China, Bt-transgenic cotton was approved for commercial use in the Yellow River and Changjiang River regions in 1997 and 2000, respectively^20^. Today, because of the widespread uses of Bt-transgenic cotton, China has become the largest cotton producing country in the world^21^. However, studies on the effects of NPs on transgenic plants and crops are minimally documented.

In this study, we investigated the effect of CeO_2_ NPs on both conventional and Bt-transgenic cotton. Upon exposure to 100 or 500 mg·L^−1^ CeO_2_ NPs, the nutrient levels, enzyme activities, and hormone concentrations in both types of cotton indicated that NPs have adverse effects regardless of the plant type. The results provide a preliminary basis for the assessment of the safety of NPs in the sustainable development of transgenic agriculture.

## Results and discussion

### Ce distribution in the stem and leaves

**Figure 1:**
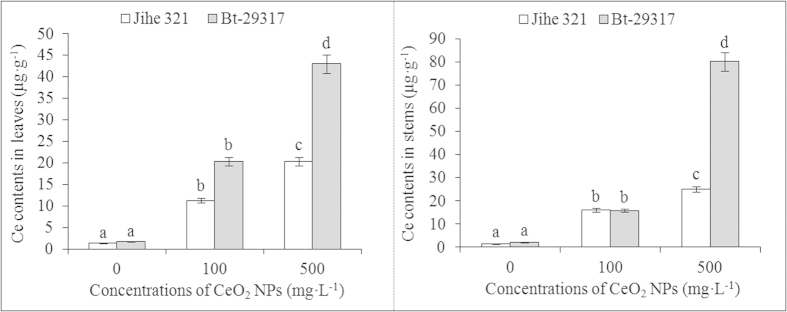
Ce contents in leaves and stems of Bt-transgenic and conventional cottons. The means are averaged from three replicates and error bars correspond to standard derivations of mean. Different small letters in the same plant indicate significant difference at p < 0.05 level between control and CeO_2_ NPs treatments, and different small letters in the same CeO_2_ NPs concentration show significant difference at p < 0.05 level between conventional and Bt-transgenic cottons.

The Ce content in the leaves and stems of old Bt-transgenic and conventional cotton plants after treatment with CeO_2_ NPs is presented in Fig. 1. The Ce content in the leaves increased with increasing doses. In the presence of 100 mg·L^−1^ CeO_2_ NPs, the Ce content in Bt-transgenic leaves was 1.8–fold higher than in conventional cotton and 12.5 fold higher than in Bt-transgenic cotton in the control group. Upon treatment with 500 mg·L^−1^ CeO_2_ NPs, the Ce content was significantly higher in the leaves of Bt-transgenic cotton than in the leaves of conventional cotton. Additionally, the Ce content in Bt-transgenic cotton was 2.1- and 26.4-fold higher than conventional cotton exposed to 500 mg·L^−1^ CeO_2_ NPs and the Bt-transgenic cotton control group, respectively. Similarly, the Ce content in the stems of both Bt-transgenic and conventional cotton increased with increasing CeO_2_ NP concentrations. When treated with 500 mg·L^−1^ CeO_2_ NPs, the Ce content in the stems of Bt-transgenic cotton was greatly elevated compared with conventional cotton. The Ce content in 500 mg·L^−1^ CeO_2_ NP-treated Bt-transgenic stems was 80.15 μg·g^−1^, which was 3.2 times the Ce content in similarly treated conventional cotton. These results suggested that CeO_2_ NPs were taken up by the cotton plants and transported to the stems and leaves from the root system. This result is consistent with previous studies stating that CeO_2_ NPs are transported to shoots from the cotton root system^15^ and that CuO NPs enter maize through the root system^22^. Lin and Xing^23^ reported that ZnO nanoparticles taken up by ryegrass roots but that few ZnO nanoparticles were transported to the shoots.

### Effect of CeO2 NPs on nutrients in xylem sap

**Figure 2:**
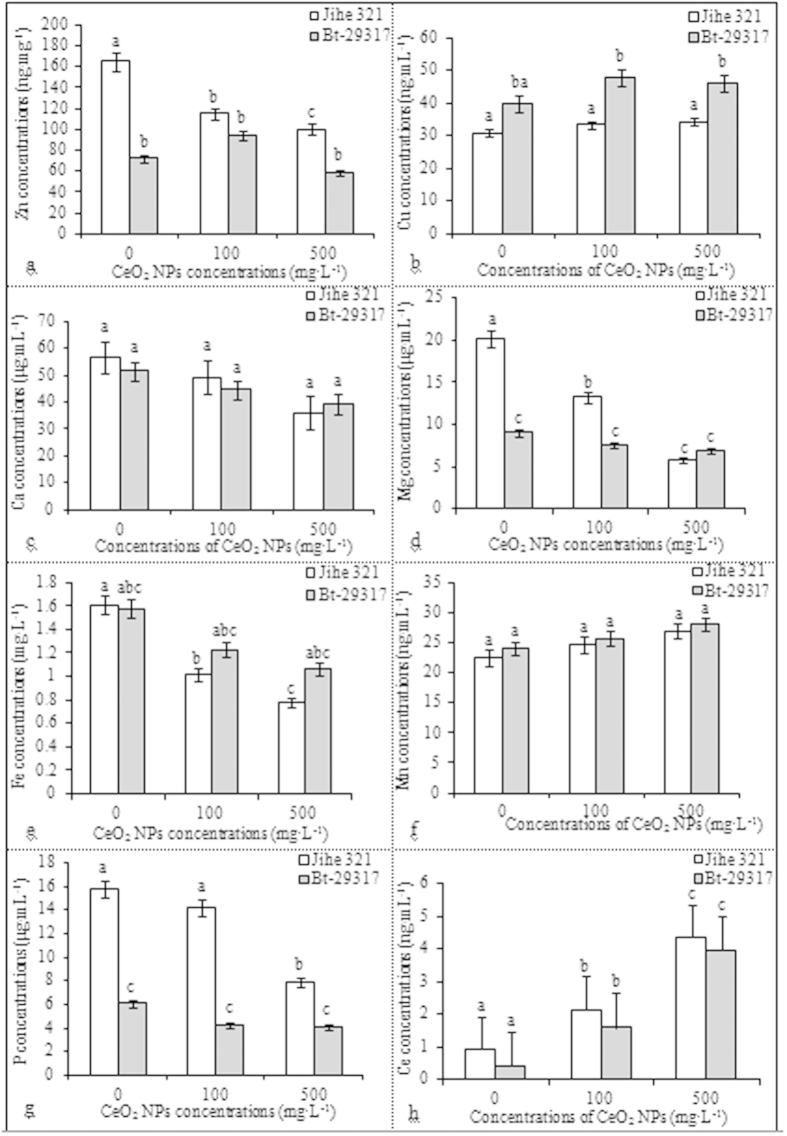
Effects of CeO_2_ NPs on the nutrient concentrations in the xylem sap. The means are averaged from three replicates and error bars corresponded to standard derivations of three values. Different small letters in the same plant indicate significant difference at p < 0.05 level between control and CeO_2_ NPs treatments, and different small letters in the same CeO_2_ NPs concentration show significant difference at p < 0.05 level between conventional and Bt-transgenic cottons.

Figure 2 presents nutrient concentrations in xylem sap from Bt-transgenic and conventional cotton plants exposed to CeO_2_ NPs. Zn, Mg, Fe, and P levels in the xylem sap after exposure to CeO_2_ NPs significantly decreased (p < 0.05), whereas the Mn concentration increased in CeO_2_ NP-treated cotton plants. The concentration of Zn in the xylem sap of conventional cotton plants decreased upon treatment with CeO_2_ NPs. As the exposure dose increased, more effects on the Zn uptake were observed. However, no significant differences in Zn content were noted between CeO_2_ NP-treated Bt-transgenic cotton and the control group. Zn levels in Bt-transgenic cotton were significantly lower (p < 0.05) than in the control group and conventional cotton, especially upon exposure to 500 mg·L^–1^ of CeO_2_ NPs. Similarly, Mg, Fe, and P concentrations in the xylem sap of conventional cotton significantly decreased (p < 0.05) after treatment with CeO_2_ NPs. Mg plays a critical role in photosynthesis; Mg is involved in chlorophyll synthesis and in the ultrastructure of chloroplasts^24^. The Mg concentration in conventional cotton treated with CeO_2_ NPs was significantly lower (p < 0.05) than in the control group (Fig. 2d). By contrast, Mg levels were significantly lower (p < 0.05) in transgenic cotton than in conventional cotton treated with 100 mg·L^−1^ CeO_2_ NPs and the control group. No significant differences were noted between the control and CeO_2_ NP-treated groups in transgenic cotton. Similarly, the Fe content in conventional cotton significantly differed (p < 0.05) between the control and the CeO_2_ NP-treated cotton samples, but no effect of CeO_2_ NPs was observed in transgenic cotton and between cultivars. The P level in the xylem sap of both transgenic and conventional cotton decreased after exposure to CeO_2_ NPs. The high CeO_2_ NP dose significantly affected (p < 0.05) the P content in the xylem sap of conventional cotton, but no significant differences in P levels were noted between the CeO_2_ NP-tested Bt-transgenic cotton and the control group. However, the P levels significantly differed (p < 0.05) between conventional and Bt-transgenic cotton in both the control and CeO_2_ NP-treated samples. These results suggested that CeO_2_ NPs did not affect P levels in the xylem sap of Bt-transgenic cotton (Fig. 2g).

No significant differences in Cu, Ca, and Mn levels in the xylem sap of both conventional and Bt-transgenic cotton were noted between the CeO_2_ NP-treated and the control samples (Fig. 2b,c,f). The effects of CeO_2_ NPs on the concentration of Cu in the xylem sap of transgenic cotton were different from those noted in conventional cotton. The Cu concentration in the xylem sap of transgenic cotton was elevated in both CeO_2_ NP-treated samples, but no effects were observed in conventional cotton. Moreover, the Cu level in Bt-transgenic cotton was significantly higher (p < 0.05) than in conventional cotton exposed to CeO_2_ NPs. These results suggest that the Cu uptake in transgenic cotton was more sensitive to CeO_2_ NPs than that in conventional cotton.

The Ce concentration in the xylem sap of both conventional and Bt-transgenic cotton increased significantly with increasing CeO_2_ NP concentrations. Minimal Ce was noted in the xylem sap of conventional and transgenic cotton plants not exposed to CeO_2_ NPs (Fig. 2h). In the CeO_2_ NP-treated samples, the concentration of Ce in the xylem sap of both conventional and transgenic cotton increased significantly, indicating that CeO_2_ NPs can be absorbed by the root and transported to the shoot via xylem sap. This result is supported by previous studies by Zhang *et al.*^25^ and Li *et al.*^15^ that indicate that CeO_2_ NPs entered the roots and were subsequently transported to the shoots of cucumber and cotton, respectively. The presence of Ce in plant tissues is further demonstrated using transmission electron microscopy (TEM).

### CeO_2_ NPs observed in xylem sap

**Figure 3:**
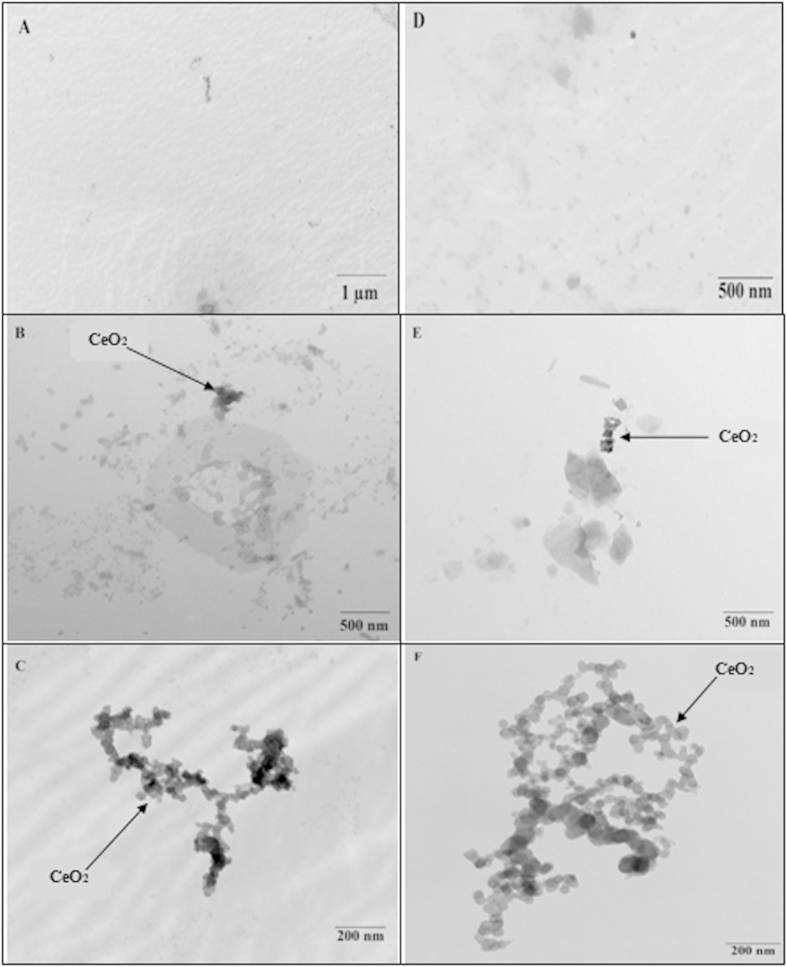
TEM images of conventional and transgenic cotton xylem sap treated with different concentration of CeO_2_ NPs. A-B-C and D-E-F are TEM images of non-transgenic and Bt-transgenic cotton under 0, 100 and 500 mg·L^−1^ CeO_2_ NPs treatments, respectively.

We observed CeO_2_ NPs in the xylem sap of both transgenic and conventional cotton plants (Fig. 3c,f), suggesting that the CeO_2_ NPs were transported from the roots to the shoots via xylem sap. Many studies report that NPs are transferred to the shoots from the root system^25^. These results suggested that CeO_2_ NPs were taken up by cotton plants. Similarly, Li *et al.*^15^ reported that a higher concentration of Ce was observed in CeO_2_ NP-treated shoots than in the control group, but our results demonstrate that CeO_2_ NPs were transported and aggregated to form clusters.

### Effect of CeO_2_ NPs on enzyme activities

**Figure 4:**
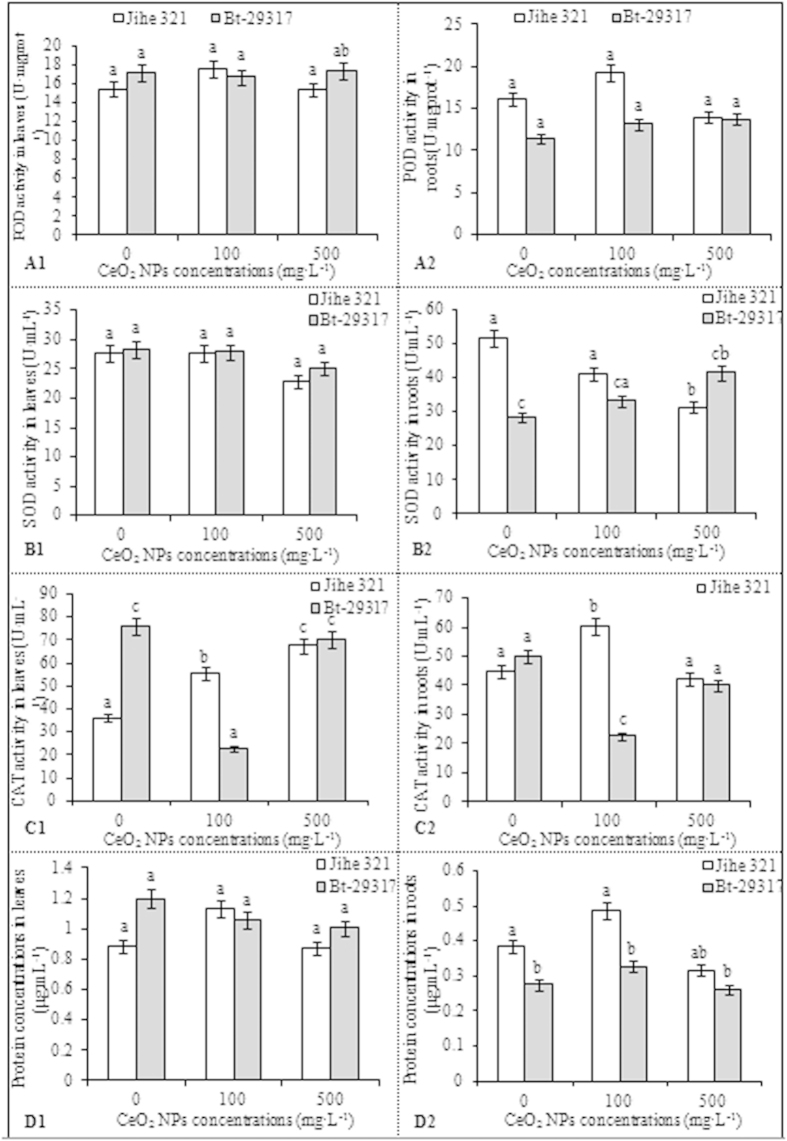
Effects of CeO_2_ NPs concentrations on the enzymes activities of conventional and transgenic cottons. A1, B1, C1, D1 and A2, B2, C2, D2 were POD, SOD, CAT activities and protein concentration in the leaves and roots of non-transgenic and Bt-transgenic cottons, respectively. The mean is averaged from three replicates and error bars correspond to standard derivation of mean. Different small letters in the same plant indicate significant difference at p < 0.05 level between control and CeO_2_ NPs treatments, and different small letters in the same CeO_2_ NPs concentration indicate significant difference at p < 0.05 level between conventional and Bt-transgenic cottons.

No significant differences in root peroxidase (POD) activities, leaf superoxide dismutase (SOD) activities, and protein concentration were noted in either Bt-transgenic or conventional cotton between the CeO_2_ NP-treated and control samples as well as between cultivars exposed to similar CeO_2_ NP concentrations (Fig. 4). POD activity was significantly higher in the leaves (p < 0.05) of Bt-transgenic cotton than in conventional cotton upon exposure to 500 mg·L^−1^ CeO_2_ NPs. Fig. 4 (B1, B2) indicates no significant difference in SOD activity in the leaves and roots of Bt-transgenic cotton and the leaves of conventional cotton for the CeO_2_ NP-treated and control groups. Upon exposure to 500 mg·L^−1^ CeO_2_ NPs, SOD activity in the roots of conventional cotton significantly differed (p < 0.05) from that of the control group. In addition, SOD activity in Bt-transgenic cotton roots was significantly lower (p < 0.05) than in conventional cotton without CeO_2_ NP treatment. However, no significant difference in root SOD activity was noted between conventional and Bt-transgenic cotton plants upon CeO_2_ exposure. The effects of CeO_2_ NPs on catalase (CAT) activity in the leaves and roots of Bt-transgenic and conventional cotton are presented in Fig. 4 (C1, C2). Upon CeO_2_ exposure, CAT activity in the leaves of conventional cotton plants significantly differed (p < 0.05) from that of the control group. However, a significant difference (p < 0.05) in the CAT activity in the leaves and roots of Bt-transgenic cotton was noted between samples treated with 100 mg·L^−1^ CeO_2_ NPs and the control group. By contrast, the CAT activity in the leaves of conventional cotton plants was lower than that of Bt-transgenic cotton in the control group, but the levels were obviously higher (p < 0.05) than in Bt-transgenic cotton treated with 100 mg·L^−1^ CeO_2_ NPs. Similar results were observed regarding root CAT activity upon exposure to 100 mg·L^−1^ CeO_2_ NPs. These results are consistent with the previous study by Ali^26^, who investigated the effects of nanoparticles on *Triticum aestivum*. According to Zhao *et al.*^27^, reduced CAT activity was noted in corn plants grown in organic soil treated with 400 mg·kg^−1^ ZnO NPs. Arnab Mukherjee^28^ reported decreased CAT activity in green peas (*Pisum sativum L*) treated with ZnO NPs.

The protein concentration in the leaves and roots of both conventional and Bt-transgenic cotton was not affected upon exposure to CeO_2_ NPs compared with the control group (Fig. 4 D1, D2). A significant difference (p < 0.05) in the root protein concentrations were noted between conventional and Bt-transgenic cotton samples treated with 100 mg·L^−1^ CeO_2_ NPs and control groups. Root protein concentrations was higher in conventional cotton than in Bt-transgenic cotton for both control groups and samples exposed to CeO_2_ NPs (Fig. 4 D2). These results indicated that CeO_2_ NPs did not affect protein concentrations in the leaves and roots of both conventional and Bt-transgenic cotton plants. This finding differs from previous studies by Salama^29^, who reported the effects of silver nanoparticles on the protein content of common bean (*Phaseolus vulgaris*) and corn (*Zea mays*). This finding suggests that various nanoparticles have different effects on the protein content of different plants.

### Effects of CeO_2_ NPs on hormone concentration

**Figure 5:**
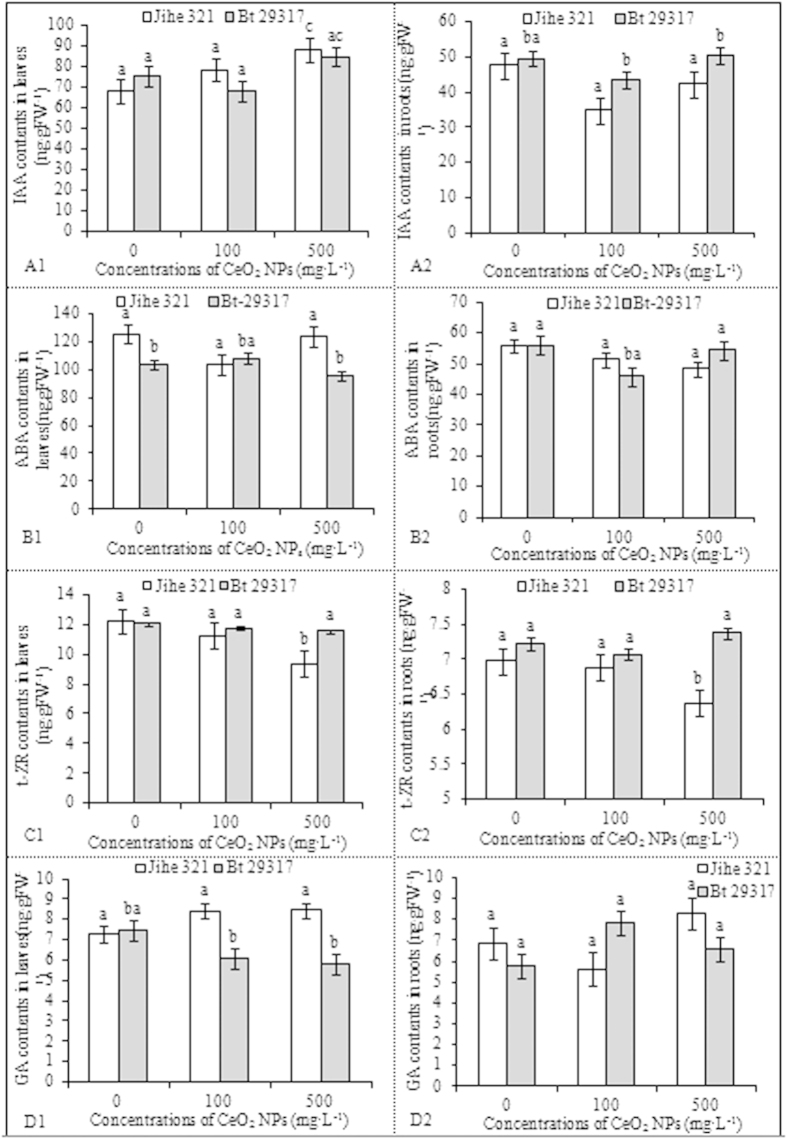
Effects of CeO_2_ NPs concentrations on contents of IAA, ABA, t-ZR and GA in the leaves and roots of Bt-transgenic and non-transgenic cotton. A1, B1, C1, D1 and A2, B2, C2, D2 were IAA, ABA, t-ZR and GA contents in the leaves and roots, respectively. The mean is averaged from three replicates and error bars correspond to standard derivation of mean. Different small letters in the same plant indicate significant difference at p < 0.05 level between control and CeO_2_ NPs treatments, and different small letters in the same CeO_2_ NPs concentration indicate significant difference at p < 0.05 level between conventional and Bt-transgenic cottons.

Figure 4 (A1, A2) indicates that the IAA content in the leaves and roots of Bt-transgenic cotton did not significantly different between the CeO_2_ NP-treated samples and the control groups. However, IAA levels in the leaves of conventional cotton treated with 500 mg·L^−1^ CeO_2_ NPs were 1.29–fold higher (p < 0.05) than in the control group. Additionally, root IAA levels of Bt-transgenic cotton exposed to 100 and 500 mg·L^−1^ CeO_2_ NPs were 1.25- and 1.19-fold higher (p < 0.05), respectively, than those of conventional cotton, whereas CeO_2_ NPs were not observed to affect IAA in the leaves of Bt-transgenic and conventional cotton. Similarly, no significant difference was noted in the ABA content of the leaves of conventional or Bt-transgenic cotton treated with CeO_2_ NPs compared with the control group. Upon exposure to 100 mg·L^−1^ CeO_2_ NPs, ABA levels in the roots of Bt-transgenic cotton were 81.98% lower (p < 0.05) than that of the control group; values were not reported for treatment at 500 mg·L^−1^ (Fig. 5 B1, B2). By contrast, trans-zeatinriboside (t-ZR) content in the leaves and roots of Bt-transgenic cotton was not affected by CeO_2_ NPs compared with the control group. The t-ZR content in the leaves and roots of conventional cotton exposed to 500 mg·L^−1^ CeO_2_ NPs was 76.57% and 91.26% lower (p < 0.05), respectively, than that in the leaves and roots of the control group. In addition, the t-ZR content in conventional cotton leaves and roots was 97.31% and 86.43% (p < 0.05), respectively, than in the leaves and roots of Bt-transgenic cotton exposed to 500 mg·L^−1^ CeO_2_ NPs (Fig. 5 C1, C2). This finding suggests that high concentrations of CeO_2_ NPs affect t-ZR content in conventional cotton but not Bt-transgenic cotton. No significant difference was observed regarding gibberellic acid (GA) content in the leaves and roots of conventional and Bt-transgenic cotton plants from the CeO_2_ NP-treated samples and the control groups (Fig. 5 D1, D2). However, the leaf GA content in conventional cotton was considerably higher (p < 0.05) than in Bt-transgenic cotton upon CeO_2_ NP treatment. Furthermore, IAA and ABA concentrations in the leaves were 2-fold higher than in the roots of both Bt-transgenic and conventional cotton plants, whereas the t-ZR concentrations were 1.5-fold higher in the leaves than in the roots. Leaf GA content was similar to that observed in the roots. These results indicate that hormone concentrations were higher in leaves than in roots under similar CeO_2_ NP concentrations. This result suggests that CeO_2_ NPs exert different effects on hormones and affect different pant regions in Bt-transgenic and conventional cotton plants.

### TEM images of CeO_2_ NP distribution in the leaves, stems, and roots

**Figure 6:**
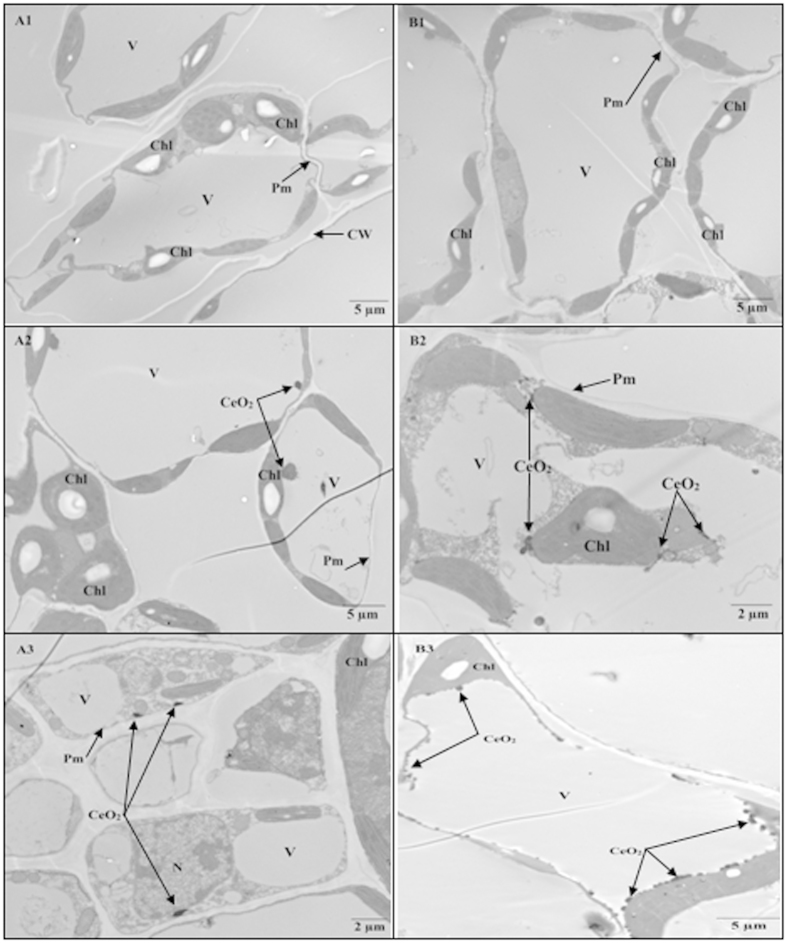
TEM images of conventional and Bt-transgenic cotton leaves. A1-A2-A3 and B1-B2-B3 are TEM images of conventional and Bt-transgenic cottons under 0, 100 and 500 mg·L^−1^ CeO_2_ NPs treatments, respectively. Chloroplast (Chl), Vacuole (V), Plasma membrane (Pm), Nucleus (N) and Cell wall (CW).

CeO_2_ NPs were observed in the leaf cells of both conventional and transgenic cotton and more CeO_2_ NPs were noted in the leaves of transgenic cotton than in those of conventional cotton (Fig. 6). At 100 mg·L^−1^ CeO_2_ NPs, most CeO_2_ NPs were distributed around the chloroplast and adsorbed on the outer membrane of the chloroplast; however, fewer CeO_2_ NPs were observed on the outer membrane of chloroplasts in the leaves of conventional cotton. As the CeO_2_ NP concentration increased to 500 mg·L^−1^, more CeO_2_ NPs were adsorbed onto the outer membrane of the chloroplast in the leaves of transgenic cotton. In addition, many chloroplasts began to swell, rupture, and exhibit deformities. In contrast, chloroplasts in the leaves of conventional cotton exhibited no obvious change. These observations are consistent with the conclusion by Li *et al.*^15^ that Bt-transgenic cotton is more sensitive to CeO_2_ NPs than is conventional cotton. All of these data indicate that chloroplasts are the most vulnerable organelle in the leaves and that the chloroplasts of transgenic cotton are more vulnerable than those of conventional cotton.

**Figure 7:**
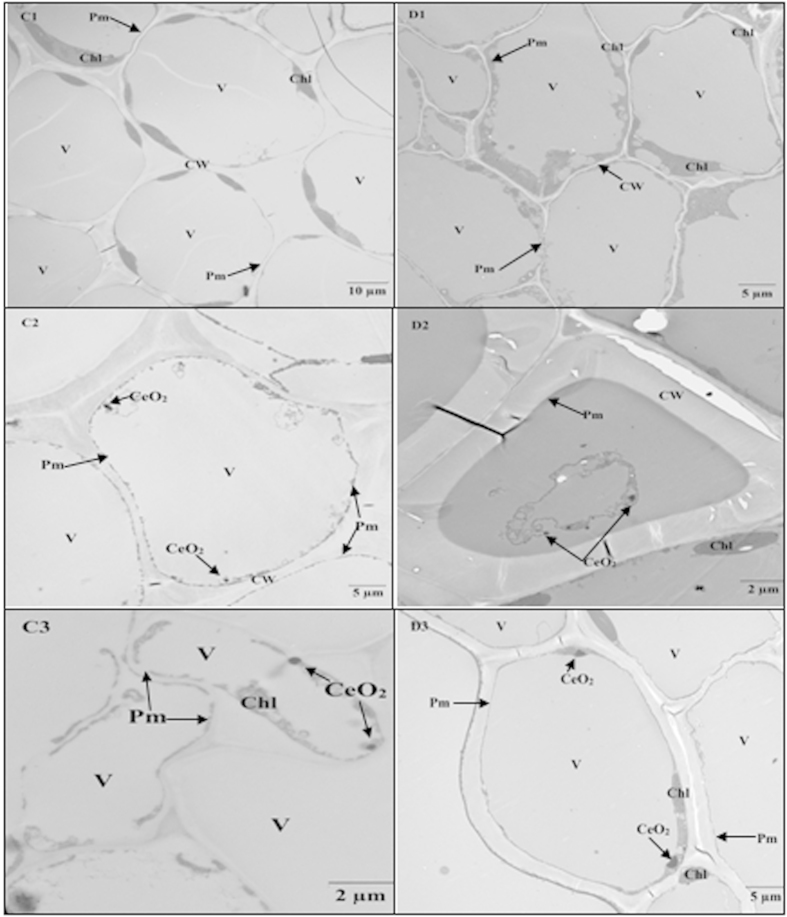
TEM images of conventional and Bt-transgenic cotton stems. C1-C2-C3 and D1-D2-D3 are TEM images of conventional and Bt-transgenic cotton under 0, 100 and 500 mg·L^−1^ CeO_2_ NPs treatments, respectively. Chloroplast (Chl), Vacuole (V), Plasma membrane (Pm), Nucleus (N), Cell wall (CW).

Numerous vascular bundle fragments were noted in both conventional and transgenic cotton exposed to 100 mg·L^−1^ CeO_2_ NPs. Compared with the CeO_2_ NP distribution on the inner wall of the vascular bundles of conventional cotton, fewer NPs were observed in transgenic cotton (Fig. 7 C2, D2). With 500 mg·L^−1^ CeO_2_ NPs, the vascular bundles in conventional cotton were deformed and damaged (Fig. 7 C3, D3). These data imply that most CeO_2_ NPs were detained in vascular bundles in conventional cotton, whereas most CeO_2_ NPs were transported into the leaves of transgenic cotton.

**Figure 8:**
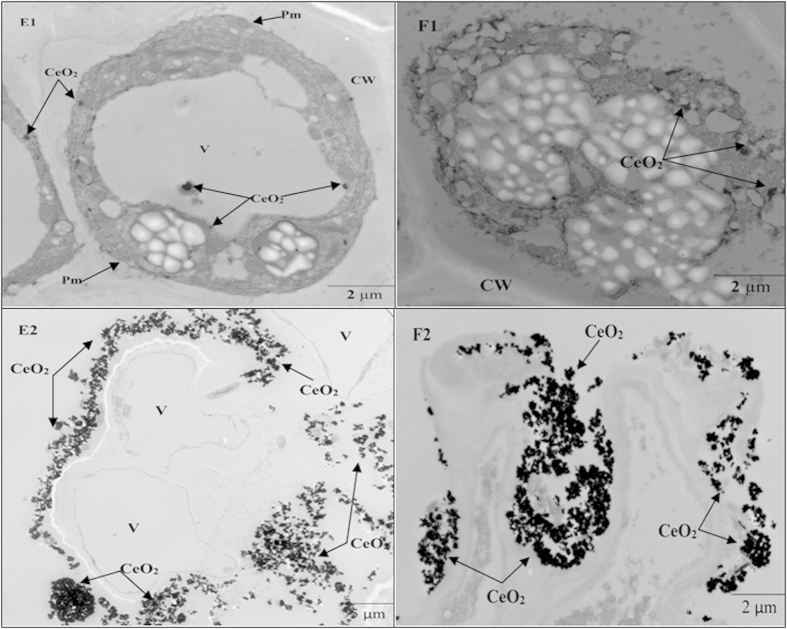
TEM images of conventional and Bt-transgenic cotton roots. E1-E2 and F1-F2 are TEM images of conventional and Bt-transgenic cotton under 100 and 500 mg·L^−1^ CeO_2_ NPs treatments, respectively. Chloroplast (Chl), Vacuole (V), Plasma membrane (Pm) and Cell wall (CW).

Figure 8 presents TEM images of conventional and Bt-transgenic cotton roots under various treatments. The adsorption of CeO_2_ NPs into the root cells and CeO_2_ NP aggregation on the root surface of both conventional and Bt-transgenic cotton were evident, and CeO_2_ NP aggregation increased as the CeO_2_ NPs exposure dose reached 500 mg·L^−1^ (Fig. 8 E2, E3, F2, F3). Li *et al.*^15^ reported that CeO_2_ NPs were observed in the roots of Bt-transgenic cotton and its parental non-transgenic cotton based on TEM. In studies by Jose *et al.*^30^, synchrotron microfocused X-ray fluorescence (μ-XRF) and micro X-ray absorption near-edge structure (μ-XANES) analyses were performed to determine the presence of CeO_2_ NPs in soybean (*Glycine max*) tissues. X-ray absorption spectroscopy (XAS) was used to demonstrate the uptake of CeO_2_ NPs into alfalfa (*Medicago sativa*), corn (*Zea mays*), cucumber (*Cucumis sativus*), and tomato (*Lycopersicon esculentum*)^31^.

## Conclusions

CeO_2_ NPs were absorbed, transported by the xylem sap, and distributed to the roots, stems, and leaves of both conventional cotton and transgenic cotton. CeO_2_ NPs significantly reduced the absorption of Zn, Mg, Fe, and P in the xylem sap of conventional cotton but increased Mn absorption. SOD activity in the roots and CAT activity in the leaves and roots of Bt-transgenic cotton plants were affected by low CeO_2_ NP concentrations. In addition, the root IAA content was obviously higher (p < 0.05) in Bt-transgenic cotton than in conventional cotton upon CeO_2_ NP treatment; the ABA content in the roots of Bt-transgenic cotton treated with 100 mg·L^−1^ CeO_2_ NPs was lower than that of the control group, but CeO_2_ NPs had no effect on GA and t-ZR content in the leaves and roots of Bt-transgenic cotton.

Most CeO_2_ NPs aggregated on the external surface of chloroplasts, which were swollen and ruptured, especially in Bt-transgenic cotton. This phenomenon could result in decreased Zn, Mg, Fe, and P levels in xylem sap because ruptured chloroplasts can release these elements, which are components of chloroplasts or chlorophyll. In addition, numerous fragments emerged in vascular bundles as their structure was destroyed. This destruction was more evident in transgenic cotton than in conventional cotton. The endocytosis of CeO_2_ NPs was observed by TEM on the surface of the root of transgenic cotton; this observation may be indicative of the mechanism by which CeO_2_ NPs entered the plant cells. The presence of CeO_2_ NPs on the surface and in the internal cells of the root is dependent on the CeO_2_ NP concentration.

## Methods

### Sample preparation and experiments

**Figure 9:**
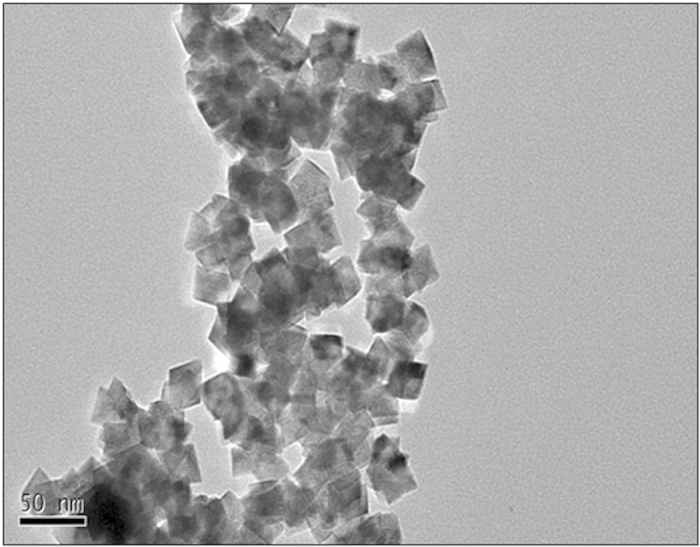
TEM image of CeO_2_ nanoparticles.

CeO_2_ NPs and other chemicals were purchased from Shanghai Hufeng Bioscience Technology Company (Shanghai, China) and Beijing Chemical Plant (Beijing, China). Experiments and samples were performed according to previous studies by Li *et al.*^15^, who investigated the effects of CeO_2_ NPs on Bt-transgenic cotton at different concentrations (0, 10, 100, 500, and 2000 mg·L^−1^). The results indicate that root biomass and nutrient uptake in Bt-transgenic cotton were affected by low concentrations of CeO_2_ NPs (100 and 500 mg·L^−1^) in contrast to conventional cotton. In addition, based on a preliminary experiment involving various concentrations of CeO_2_ NPs (0, 10, 100, 500, 1000, and 2000 mg·L^−1^) (please see the supplementary information), three concentrations of CeO_2_ were selected (0, 100, and 500 mg·L^−1^ CeO_2_ NPs). Bt-transgenic cotton (Bt-29317) and conventional cotton (Jihe 321) were germinated in sterilised, moist sand, and two seedlings were then transplanted to 4.0-L pots containing 3.0 L of nutrient solution. After one week, the nutrient solution was amended with 0, 100, and 500 mg·L^−1^ CeO_2_ NPs. The average diameter of the CeO_2_ NPs was 10 ± 3.2 nm, as measured by TEM before use in the experiments (please see the supplementary information). TEM also indicated that the CeO_2_ NPs exhibited an octahedral morphology and easily agglomerated (Fig. 9). The cotton plants were grown in the greenhouse of the China Agricultural University from May to June of 2014, and three replicates were performed for each treatment.

### Determination of nutrients and Ce content in xylem sap

Xylem sap was collected 2 cm below the cotyledon with a surgical blade when the plants developed 8 to 9 leaves. The harvested samples were rinsed with deionised water to remove any disrupted and residual cell. The cut stem was connected to a centrifugal tube by a flexible silicon tube (15 mm length, 2 mm internal diameter). Xylem sap was collected for 15 min by a pressure chamber (0.2–0.3 MPa)^23^. The Ce, Cu, and Zn content in xylem sap was determined using inductively coupled plasma mass spectrometry (ICP-MS) (DRC-II). The Ca, Mg, Fe, Mg, and P content was determined by inductively coupled plasma – atomic emission spectroscopy (ICP-AES) (iCap 6000).

### Measurement of enzyme activities and protein content

After washing with tap and deionised water, fresh leaves and roots were separately harvested, cut, and mixed. Approximately 0.2 g of fresh sample was packed with aluminium paper and soaked into liquid nitrogen to protect the enzyme activities before storing at 4 °C. Each 0.2 g sample was homogenised in 1.8 mL of normal saline solution to generate a 10% sample compound solution. Then, the tissues were finely ground and centrifuged at 3500 RPM for 10 min. The extract was used to measure CAT, SOD, and POD activities as previously described by Xu *et al.*^32^ and Cho *et al.*^33^. CAT activity was measured by monitoring the degradation of H_2_O_2_ at 240 nm^32^ and SOD activity was determined by measuring inhibition of the photochemical reduction of nitrobluetetrazolium at 560 nm^34^. POD activity in the extract was measured by following the formation of guaiacol dehydrogenation products, as determined by an increase in absorbance at 470 nm^35^. Protein concentrations were determined using bovine serum albumin (BSA) as the standard in the Bradford assay^36^.

### Hormones

The extraction and purification of ABA, IAA, t-ZR, and GA were performed according to the previous studies of He *et al.*^37^. Approximately 0.2 g of fresh leaves and root samples was separately homogenised in 2 mL of 80% methanol (containing 40 mg·L^−1^ butylated hydroxy toluene) and stored at –20 °C for 48 h. The solution was then centrifuged at 3500 RPM for 15 min. The precipitates were re-suspended in 1 mL of 80% methanol at –20 °C for 16 h. C_18_ Sep-Pak cartridges (Waters, Milford, USA) were applied for the purification of the combined extracts. The samples were then evaporated under vacuum to remove the organic solvent and were dissolved in 2.0mL of TBS buffer (Tris-buffered saline; 50 mM Tris, pH 7.8, 1 mM MgCl_2_, 10 mM NaCl, 0.1% Tween, 0.1% gelatin). ABA, IAA, t-ZR, and GA levels were determined by enzyme-linked immunosorbent assay (ELISA) kits using monoclonal antibodies (Phytodetek, Agdia, Elkhart, IN, USA). Absorbance was recorded at 450 nm.

### Transmission Electron Microscopy

The plant tissues for transmission electron microscopy (TEM) observation were collected from Bt-transgenic and conventional cotton after two weeks of CeO_2_ NP exposure. The roots, stems, and leaves were thoroughly washed with deionised water, prefixed in 2.5% glutaraldehyde, dehydrated in a graded acetone series, and embedded in Spurr’s resin. The samples were sectioned for TEM analysis using a microtome JEM-1230 (JEOL, Ltd., Japan) with a diamond knife^25^,^38^,^39^.

### Data analysis

Each treatment was performed in triplicate, and the results presented in the figures are the mean ± standard deviation (SD). Statistical analysis was performed using one-way analysis of variance (ANOVA) followed by Tukey’s HSD test and an independent samples t-test using SPSS 22.0 software. A confidence interval of 95% (p < 0.05) was considered significant in all cases.

## Additional Information

**How to cite this article**: Nhan, L. V. *et al.* Phytotoxic Mechanism of Nanoparticles: Destruction of Chloroplasts and Vascular Bundles and Alteration of Nutrient Absorption. *Sci. Rep.* **5**, 11618; doi: 10.1038/srep11618 (2015).

## Supplementary Material

Supplementary Information
