# Supplementary material for: Phytotoxic Mechanism of Nanoparticles: Destruction of Chloroplasts and Vascular Bundles and Alteration of Nutrient Absorption
Source: Sci Rep. 2015 Jun 25;5:11618. doi: 10.1038/srep11618 (PMC4479828; doi:10.1038/srep11618)
Supplement: Supplementary Information [file srep11618-s1.doc]

**Phytotoxic Mechanism of Nanoparticles: Destruction of Chloroplasts and Vascular Bundles and Alteration of Nutrient Absorption**

(SREP-14-14674)

Le Van Nhan^1,2^, Chuanxin Ma^3^, Yukui Rui^1,3*^, Shutong Liu^1^, Xuguang Li^1^, Baoshan Xing^3^, Liming Liu^1^

^1^ College of Resources and Environmental Sciences, China Agricultural University, Beijing 100193, China.

^2^ Research Institute for Aquaculture No.1, Tu Son, BacNinh222260, Vietnam.

^3^Stockbridge School of Agriculture, University of Massachusetts, Amherst, MA 01003, USA.

***Corresponding author:** RuiYukui, Email: *ruiyukui@163.com*; Tel: 8610-62733470.

Number of Figures: 2

Number of Tables: 0

Number of Pages: 3

**1. Inhibitory concentration selection of CeO_2_ NPs**

In order to find out the best concentration of CeO_2_ NPs for this study, a wide range of exposure doses (0-2000 mg·L^-1^) of CeO_2_ NPs was selected to expose to both conventional and Bt-transgenic cottons. Fig. S2 showed the effects of CeO_2_ NPs on biomass and shoot heights of conventional and transgenic cottons. In Fig. S2A, NPs did not affect on plant growth upon exposure to 100 mg·L^-1^ CeO_2_ NPs regardless plant types. As concentration increased to 500 mg·L^-1^, total biomass was inhibited notably compared to control group. However, no significant difference of total biomass was found between 500 and 2000 mg·L^-1^ CeO_2_ NPs treatments. Interestingly, both conventional and Bt-transgenic cottons attained the similar biomass regardless of exposure doses of CeO_2_ NPs. It seemed that CeO_2_ NPs could not impact plants heights in both plant types (Fig. S2B), although shoot height of transgenic cotton was shorter than conventional cotton among all treatments. Based on the preliminary results, we used 100 and 500 mg·L^-1^ of CeO_2_ NPs for the following studies. In the other hand, Bt-transgenic and conventional cottons were investigated under different concentration exposures of CeO_2_ NPs such as 0, 10, 100, 500, and 2000 mg·L^-1^ by Li *et al*. (2014) ^[16]^. Results indicated that root biomass and nutrient uptake in Bt-transgenic cotton were affected by low concentrations of CeO_2_ NPs (100 and 500 mg·L^-1^) in contrast to conventional cotton. Therefore, three concentrations of CeO_2_ NPs (0, 100 and 500 mg·L^-1^) were selected for studies.


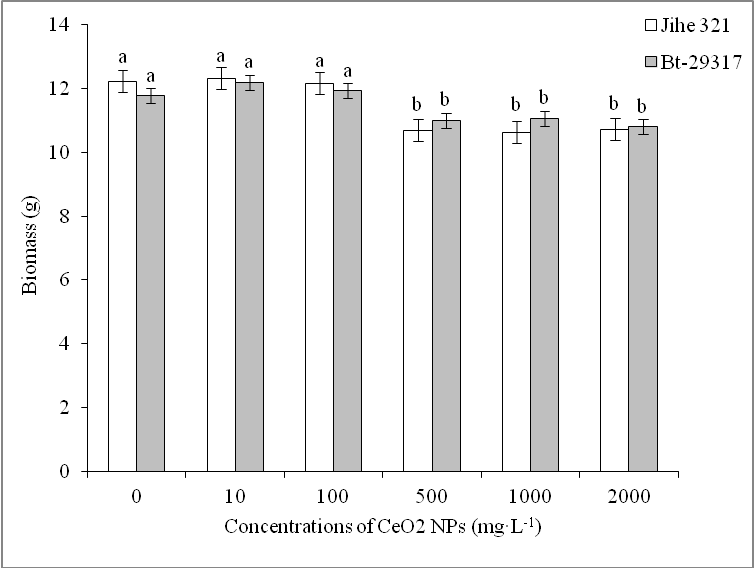


A


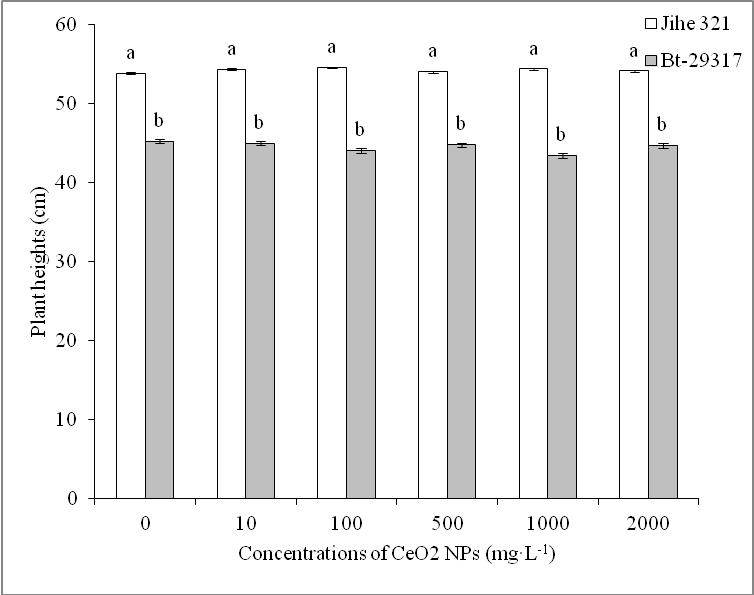


B

**Fig. 2 Effects of CeO_2_ on biomass and plant height of conventional and transgenic cottons**. The means are averaged from three replicates and error bars correspond to standard derivations of mean. Different small letters in the same plant indicate significant difference at p < 0.05 level between control and CeO_2_ NP treatments, and different small letters in the same CeO NPs concentration show significant difference at p < 0.05 level between conventional and Bt-transgenic cottons.

**2. The TEM image of CeO_2_** nanoparticles was insteaded by the clearly and better one.

| 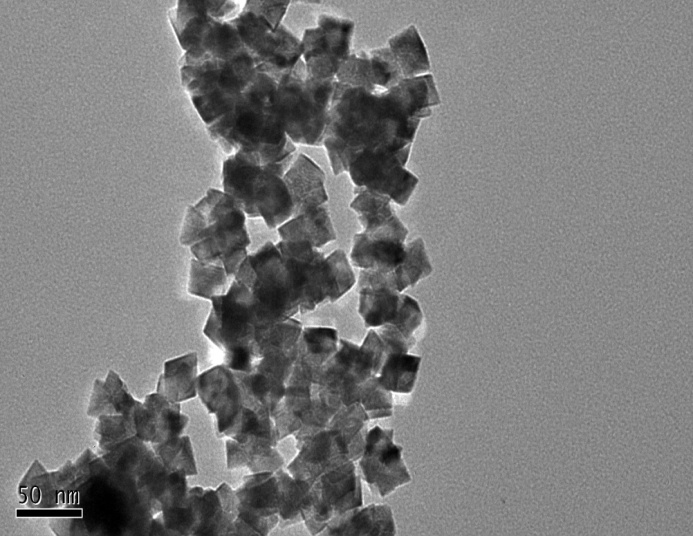**Fig. S1 TEM image of CeO_2_ NPs** |
| --- |

**3. Figure 6, 7 and 8** were supported some information such as Chloroplast (Chl), Vacuole (V), Plasma membrane (Pm) and Cell wall (CW).

| **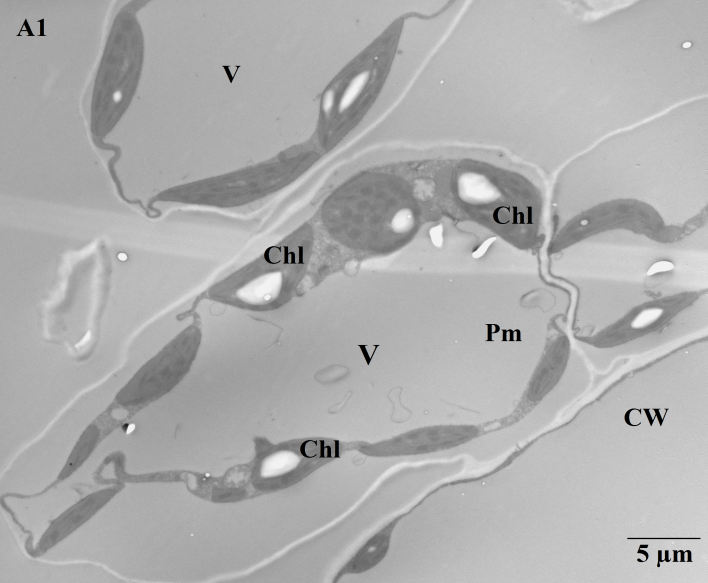** | **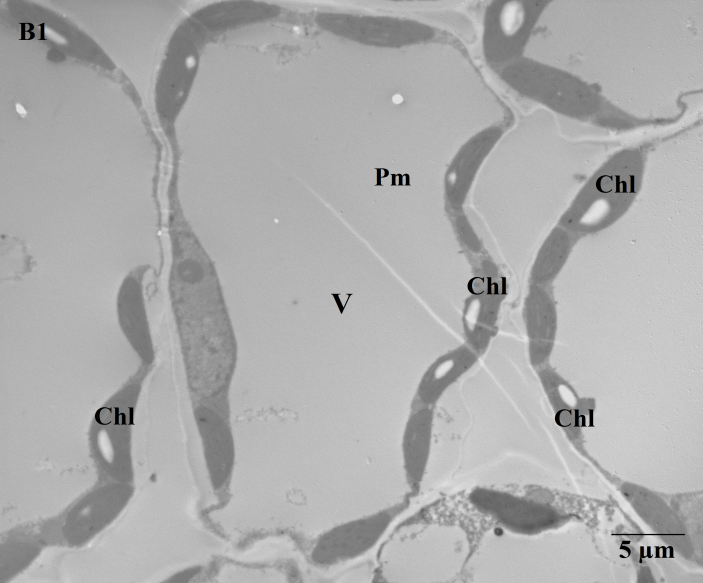** |
| --- | --- |
| **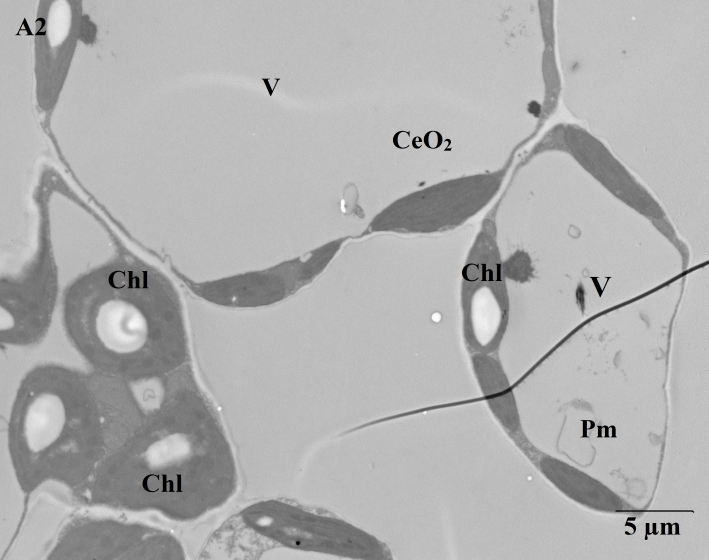** | **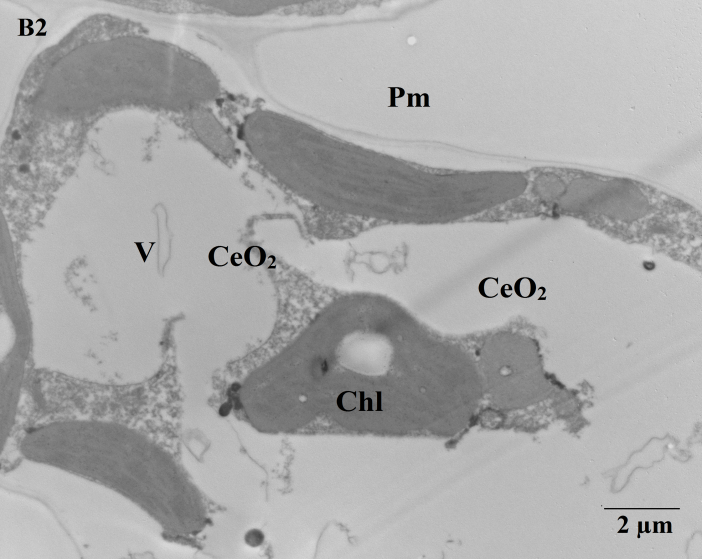** |
| **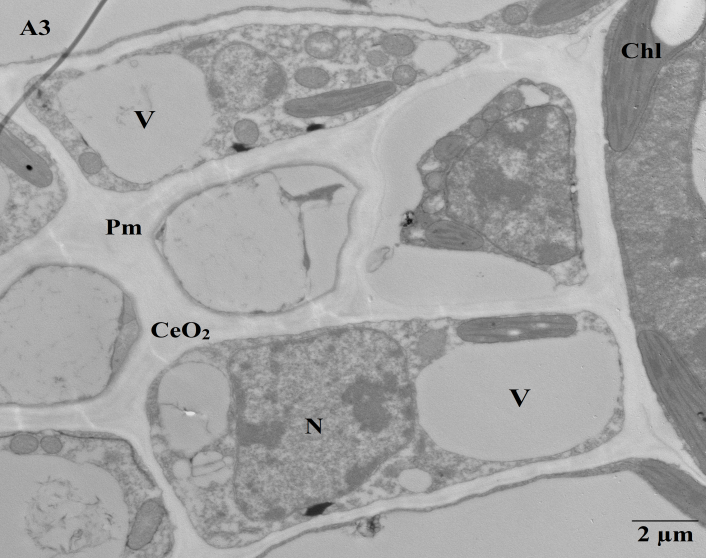** | **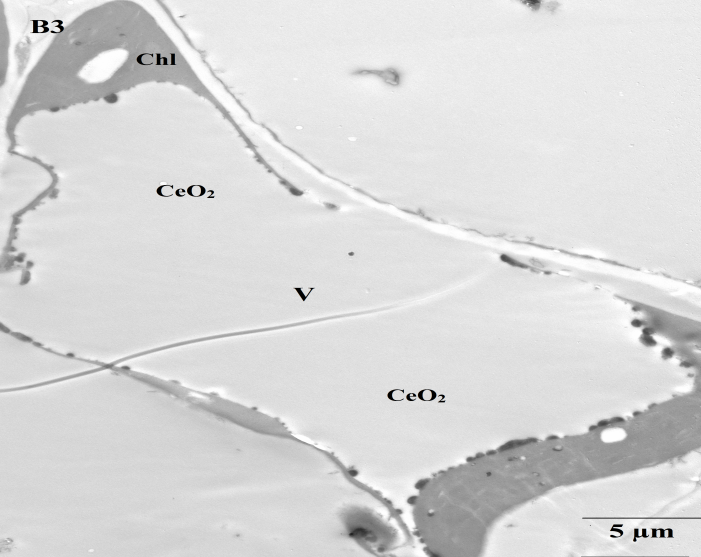** |

**Fig. 6** TEM images of conventional and Bt-transgenic cotton leaves. A1-A2-A3 and B1-B2-B3 are TEM images of conventional and Bt-transgenic cottons under 0, 100 and 500 mg·L^-1^ CeO_2_ NPs treatments, respectively. Chloroplast (Chl), Vacuole (V), Plasma membrane (Pm), Nucleus (N) and Cell wall (CW).

| **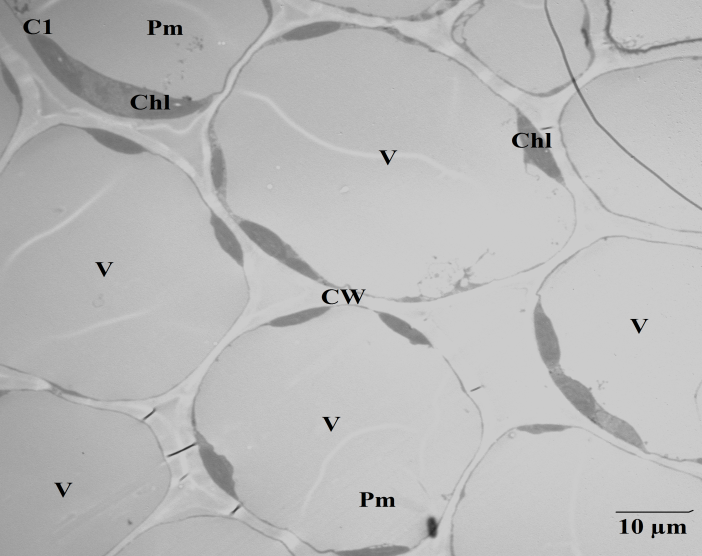** | | **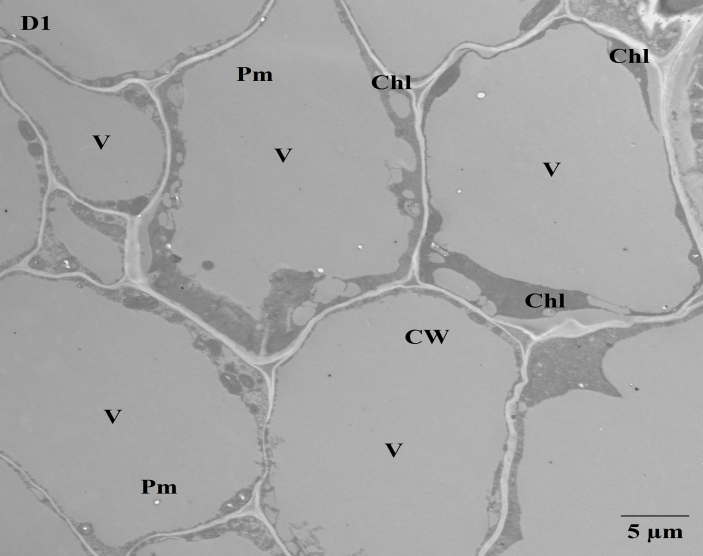** | |  |
| --- | --- | --- | --- | --- |
| **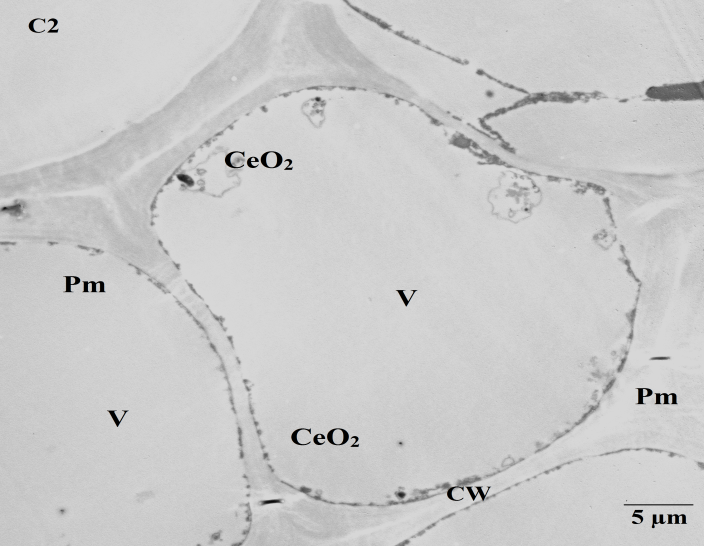** | | **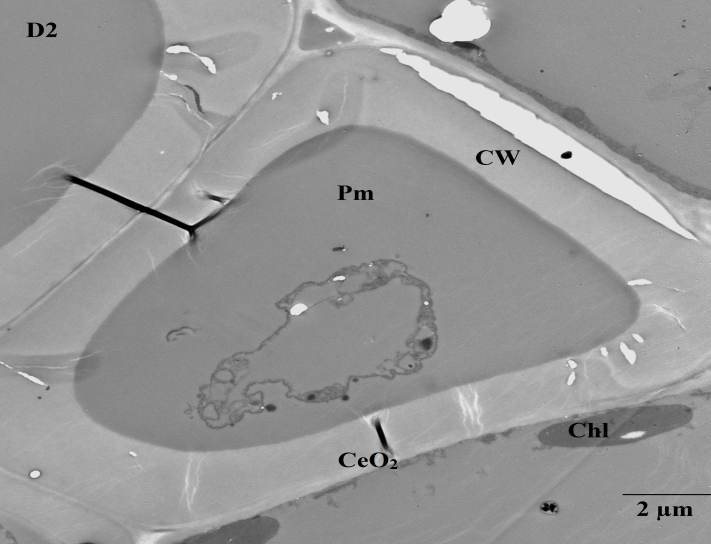** | |  |
|  | 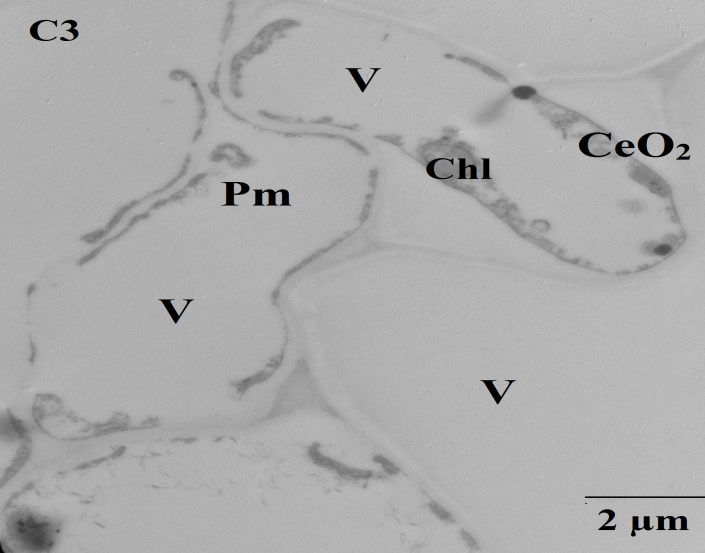 | | **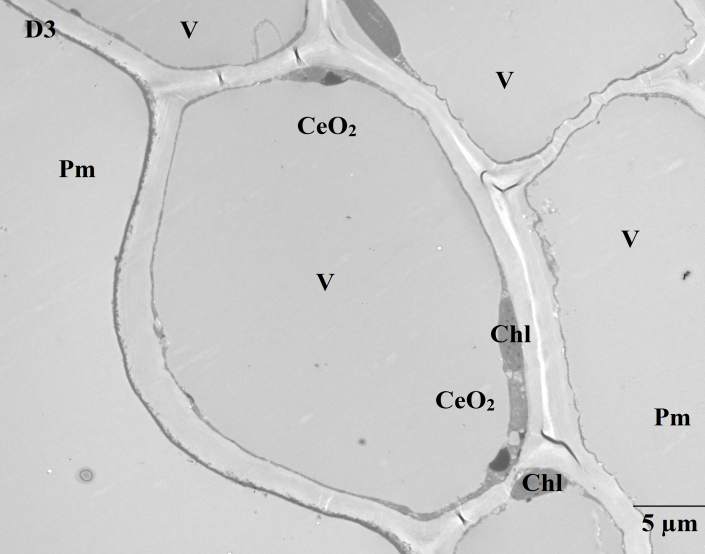** | |

**Fig. 7 TEM images of conventional and Bt-transgenic cotton stems. C1-C2-C3 and D1-D2-D3 are TEM images of conventional and Bt-transgenic cotton under 0, 100 and 500 mg·L^-1^ CeO_2_ NPs treatments, respectively.** **Chloroplast (Chl), Vacuole (V), Plasma membrane (Pm), Nucleus (N), Cell wall (CW).**

| **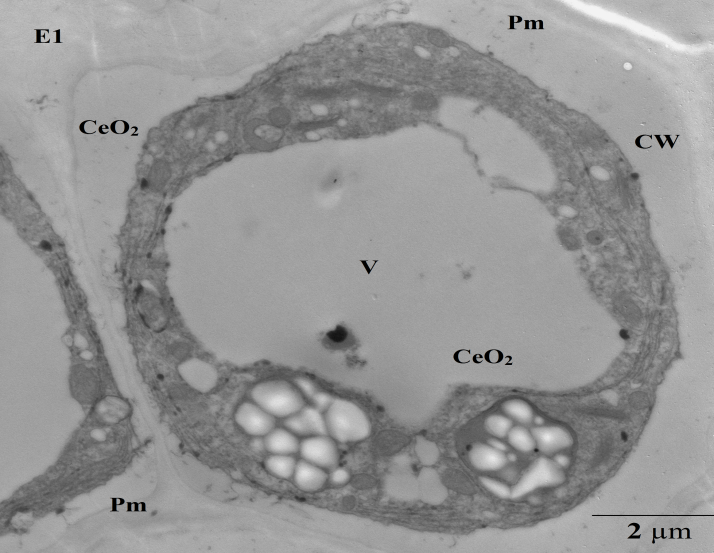** | | **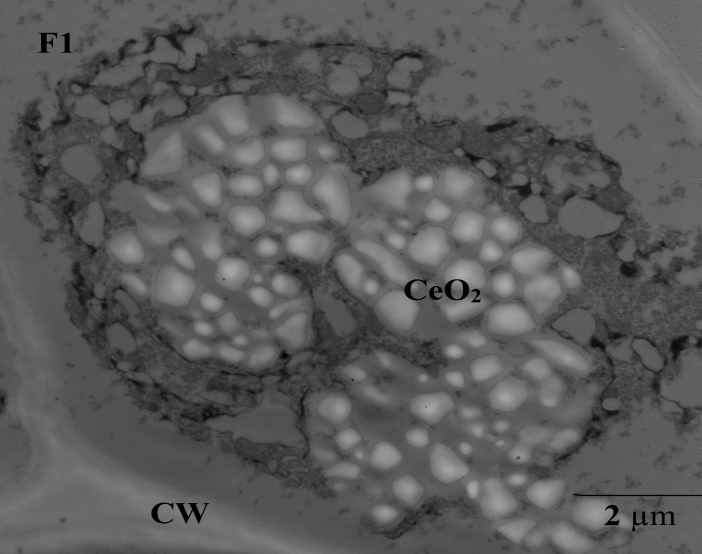** |
| --- | --- | --- |
| **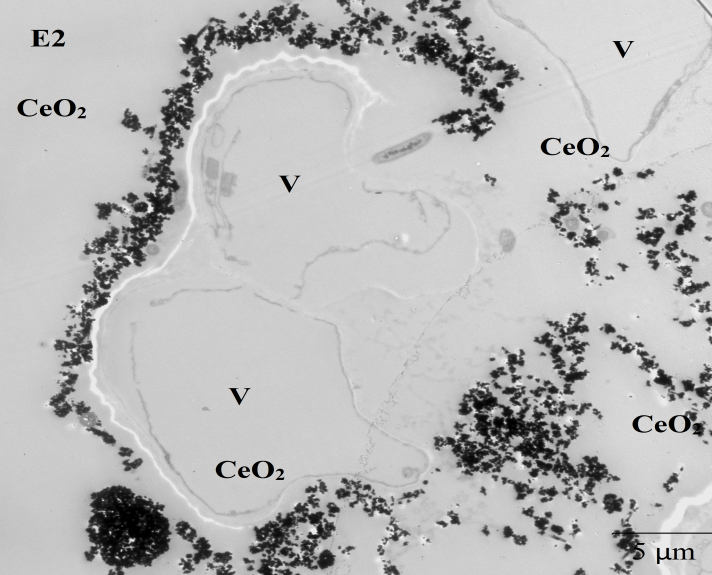** | **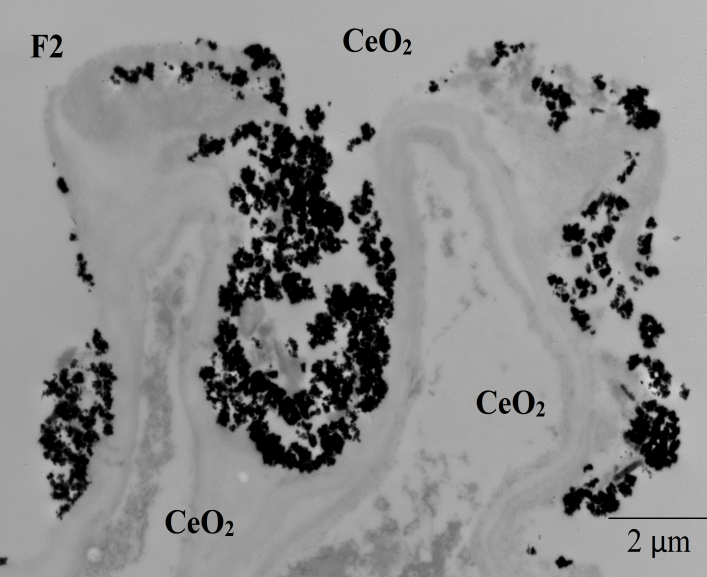** | |

**Fig. 8** TEM images of conventional and Bt-transgenic cotton roots. E1-E2 and F1-F2 are TEM images of conventional and Bt-transgenic cotton under 100 and 500 mg·L^-1^ CeO_2_ NPs treatments, respectively. Chloroplast (Chl), Vacuole (V), Plasma membrane (Pm) and Cell wall (CW).

**4. Introduction has been rewritten**

Nanomaterials have been appliedare used in many aspects of lifeprocesses and products, including wastewater treatment, food processing, catalytic materials, and biomedical products ^[1-4]^. They are used in agriculture to enhance seed germination and plant growth and to protect crops from biotic stresses, such as insects, fungi, and bacteria ^[5-8]^. However, given the unique properties of nanoparticles (NPs), living organisms in the ecosystem could suffer from oxidative stress induced by NPs ^[9-11]^. A common conclusion from recent studies was that the nanotoxicity might depend on either metal speciation or the plant species. Ma *et al*. ^[12]^ reported that exposure to 2000 mg·L^-1^ NPs from the lanthanide series, such as lanthanum oxide (La_2_O_3_), gadolinium oxide (Gd_2_O_3_), and ytterbium oxide (Yb_2_O_3_), greatly inhibits root growth in radish, tomato, rape, lettuce, wheat, cabbage, and cucumber plants. According to Lin and Xing ^[13]^, a concentration of 200 mg·L^-1^ of nano-sized Zn (zinc, 35 nm) and ZnO (zinc oxide, 20 nm) inhibited germination in ryegrass and corn, respectively. The root growth of radish, rape canola, ryegrass, lettuce, corn, and cucumber species was also inhibited upon exposure to mg·L^-1^ nano-sized Zn and ZnO ^[13]^. Silicon oxide NPs (SiO_2_) notably inhibit plant growth and the shoot and root biomasses of *Bacillus thuringiensis* (Bt)-transgenic cotton ^[14]^. Le *et al*. ^[14]^ reported that the height, shoot biomass, and root biomass of Bt-transgenic cotton were 65.39%, 68.55%, and 57.69%, respectively, of those of the control group when exposed to 2000 mg·L^-1^ of SiO_2_ NPs. Furthermore, Li *et al.* found that cerium oxide (CeO_2_) NPs disrupt the uptake of nutrient elements in Bt-transgenic cotton compared with its parental conventional cotton plant ^[5]^. Thus, the phytotoxic mechanism of nanomaterials must be thoroughly understood before application in fields.

Transgenic plants offer numerous advantages to producers, consumers, and the environment. Indeed, engineered plants are highly tolerant to pesticides and herbicides, demonstrate resistance towards pathogens caused by microorganisms, and exhibit increased production yields and enhanced biofuel content, which are considered novel and sustainable energy sources ^[16]^. Bt-transgenic cotton contains one foreign gene derived from the bacterium *Bacillus thuringiensis* that encodes the Cry toxin, which effectively kills bollworms and therefore controls yield losses ^[17-19]^. In China, Bt-transgenic cotton was approved for commercial use in the Yellow River and Changjiang River regions in 1997 and 2000, respectively ^[20]^. Today, because of the widespread uses of Bt-transgenic cotton, China has become the largest cotton producing country in the world ^[21]^. However, studies on the effects of NPs on transgenic plants and crops are minimally documented.

In this study, we investigated the effect of CeO_2_ NPs on both conventional and Bt-transgenic cotton. Upon exposure to 100 or 500 mg·L^-1^ CeO_2_ NPs, the nutrient levels, enzyme activities, and hormone concentrations in both types of cotton indicated that NPs have adverse effects regardless of the plant type. The results provide a preliminary basis for the assessment of the safety of NPs in the sustainable development of transgenic agriculture.
